# Impact of the COVID-19 Pandemic on Routine Adult Vaccination in Primary Care: A Multicenter Interrupted Time Series Analysis

**DOI:** 10.3390/vaccines14090785

**Published:** 2026-09-08

**Authors:** Begüm Yıldırım Erence, Feyzanur Erdem, Ebru Tepe, Seçil Arıca

**Affiliations:** Department of Family Medicine, Istanbul Prof. Dr. Cemil Taşcıoğlu City Hospital, Istanbul 34384, Turkey; feyzanur.erdem1@saglik.gov.tr (F.E.); ebru.tepe@sbu.edu.tr (E.T.); secil.arica@sbu.edu.tr (S.A.)

**Keywords:** vaccination, COVID-19, primary health care, interrupted time series analysis, pneumococcal vaccines, socioeconomic factors

## Abstract

**Background/Objectives:** This study evaluated changes in routine adult vaccination across the COVID-19 pandemic period in Istanbul primary care using interrupted time series (ITS) analysis, disentangling immediate from gradual effects and comparing patterns across socioeconomically distinct centers, through a multicenter retrospective analysis of routine (non-COVID-19 vaccination records). **Methods:** The full descriptive dataset comprised 33,016 adult vaccination records. After excluding 25,805 COVID-19 vaccination records, 7211 routine (non-COVID-19) vaccination records from nine family medicine units in three Istanbul training family health centers—Şişli-Gülbahar, Kağıthane, and Gaziosmanpaşa—were included in the routine vaccine and ITS analyses and were analyzed over 97 monthly observations (June 2017–August 2025), divided into three 672-working-day periods: pre-pandemic, pandemic, and post-pandemic. Segmented negative binomial ITS regression with a log (working days) offset was the primary analysis; Newey-West HAC-robust Poisson regression served as sensitivity analysis. Catchment districts were characterized using the 2022 Socioeconomic Development Ranking (SEGE-2022). **Results:** No significant immediate level change occurred in total routine vaccination at pandemic onset. Pneumococcal vaccination showed the most pronounced signal: an upward pretrend (annual slope IRR = 2.583; *p* = 0.001), a level increase at onset (IRR = 3.589; *p* = 0.013), then a rapid within-pandemic slope decline (annual slope IRR = 0.227; *p* < 0.001). Tetanus-diphtheria (Td) vaccination fell sharply at onset (IRR = 0.686; *p* = 0.002) and continued declining post-pandemic (annual slope IRR = 0.805; *p* = 0.008). Versus Gaziosmanpaşa (rank 85/973), Kağıthane (rank 38) showed the highest total routine vaccination rate (excluding COVID-19 vaccination) (IRR = 1.539; *p* < 0.001), exceeding Şişli (rank 1; IRR = 1.269; *p* < 0.001). **Conclusions:** Changes in routine adult vaccination across the pandemic period were vaccine-specific: pneumococcal vaccination surged then declined; Td vaccination showed continued post-pandemic decline. Center-level vaccination patterns were not consistently aligned with district-level socioeconomic ranking.

## 1. Introduction

Vaccination is among the most effective public health interventions, preventing an estimated 3.5–5 million deaths worldwide each year [1], yet adult vaccination coverage remains suboptimal even in high-income settings [2]. In Türkiye, childhood immunization has achieved considerable success, but adult vaccination rates fall short of targets even among recommended risk groups [3]; Istanbul physicians identify physician knowledge, reminder systems, and patient awareness as the main barriers [4]. Adult vaccines are delivered through primary care under Türkiye’s Expanded Immunization Programme, but coverage is not systematically monitored nationally [5,6].

The COVID-19 pandemic profoundly disrupted immunization systems worldwide: a 170-country analysis documented pandemic-related service disruption [7], corroborated by WHO pulse surveys across 129 countries [8,9]. Zero-dose children exceeded 8 million between 2020 and 2022, and global vaccination coverage had still not returned to pre-pandemic levels as of 2023 [10,11]. A US study of 6.5 million primary care patients found non-COVID adult vaccinations declined 4.2% in the first pandemic year and 14.2% in the second [12,13]; a 23-country survey found COVID-19 booster intent fell from 87.9% to 71.6% between 2022 and 2023 despite greater reported willingness to receive non-COVID vaccines [14]; and a systematic review confirmed broader adverse effects on trust in routine immunization [15].

Interrupted time-series studies from other settings have demonstrated substantial disruptions in routine immunization during the COVID-19 pandemic, with subsequent trajectories varying across settings and vaccine programs. In Kenya, Ngigi et al. identified pandemic-associated interruptions in routine childhood vaccination followed by relatively rapid recovery, whereas a nationwide Brazilian ITS analysis reported marked declines in childhood vaccination coverage followed by gradual post-pandemic recovery for several vaccines [16,17]. During the study period, Türkiye also implemented a nationwide mass COVID-19 vaccination program, with family health centers serving as major vaccination sites [18]. Routine adult risk-group vaccination programs, including pneumococcal, influenza, and hepatitis vaccination, continued within the national immunization framework [19]; therefore, concurrent changes in vaccination policy, vaccine supply, and service organization may have influenced recorded vaccination activity during the observation period. However, comparable evidence on routine adult vaccination remains limited, particularly from multicenter primary care settings with extended post-pandemic follow-up. This study evaluated temporal changes in routine adult vaccination across nine family medicine units in three Istanbul training family health centers, disentangling immediate from gradual pandemic effects and comparing patterns across centers with differing district-level socioeconomic development scores and rankings.

## 2. Materials and Methods

### 2.1. Study Design and Setting

This retrospective ecological interrupted time-series study of aggregate vaccination records was conducted at three training family health centers (FHCs) affiliated with Istanbul Prof. Dr. Cemil Taşcıoğlu City Hospital: Şişli-Gülbahar (Center A), Kağıthane (Center B), and Gaziosmanpaşa (Center C), comprising nine units.

Catchment-district socioeconomic development was characterized using the 2022 Socioeconomic Development Ranking of Districts (SEGE-2022), published by the Turkish Ministry of Industry and Technology, which ranks 973 districts across 56 indicators spanning demographics, employment, education, health, finance, and quality of life [20]. Center A serves Şişli (rank 1, score 6.959), Center B serves Kağıthane (rank 38, score 2.174), and Center C serves Gaziosmanpaşa (rank 85, score 1.460) (Table 1); Center C served as reference for center-level comparisons.

### 2.2. Definition of Pandemic Periods

Three periods were defined based on Türkiye’s first confirmed COVID-19 case (11 March 2020) [21]. For the primary analysis, 27 November 2022 was operationally defined as the pandemic endpoint, corresponding to the discontinuation of daily COVID-19 case reporting by the Turkish Ministry of Health [22]. WHO’s declaration of the end of the COVID-19 Public Health Emergency (5 May 2023) was the accepted international reference event [23]. Weekends and official/religious holidays were excluded so that each period comprised exactly 672 working days: pre-pandemic (30 June 2017–10 March 2020), pandemic (11 March 2020–27 November 2022), and post-pandemic (28 November 2022–8 August 2025). Equal working-day, rather than equal calendar-time, periods were used to avoid bias from unequal clinical service availability. This standardization was used as a design-level comparability measure rather than as a substitute for model-based adjustment; monthly working days were additionally retained as an offset in all ITS models to account for residual month-to-month variation in clinical operating time. Two additional sensitivity analyses were conducted to assess the robustness of the period definitions. First, the ITS models were repeated using the full available pre-pandemic period beginning in January 2017 without enforcing equal period lengths. Second, the models were repeated using 5 May 2023, when WHO declared the end of COVID-19 as a Public Health Emergency of International Concern, as the alternative pandemic endpoint [23].

### 2.3. Data Source and Eligibility

**Figure 1:**
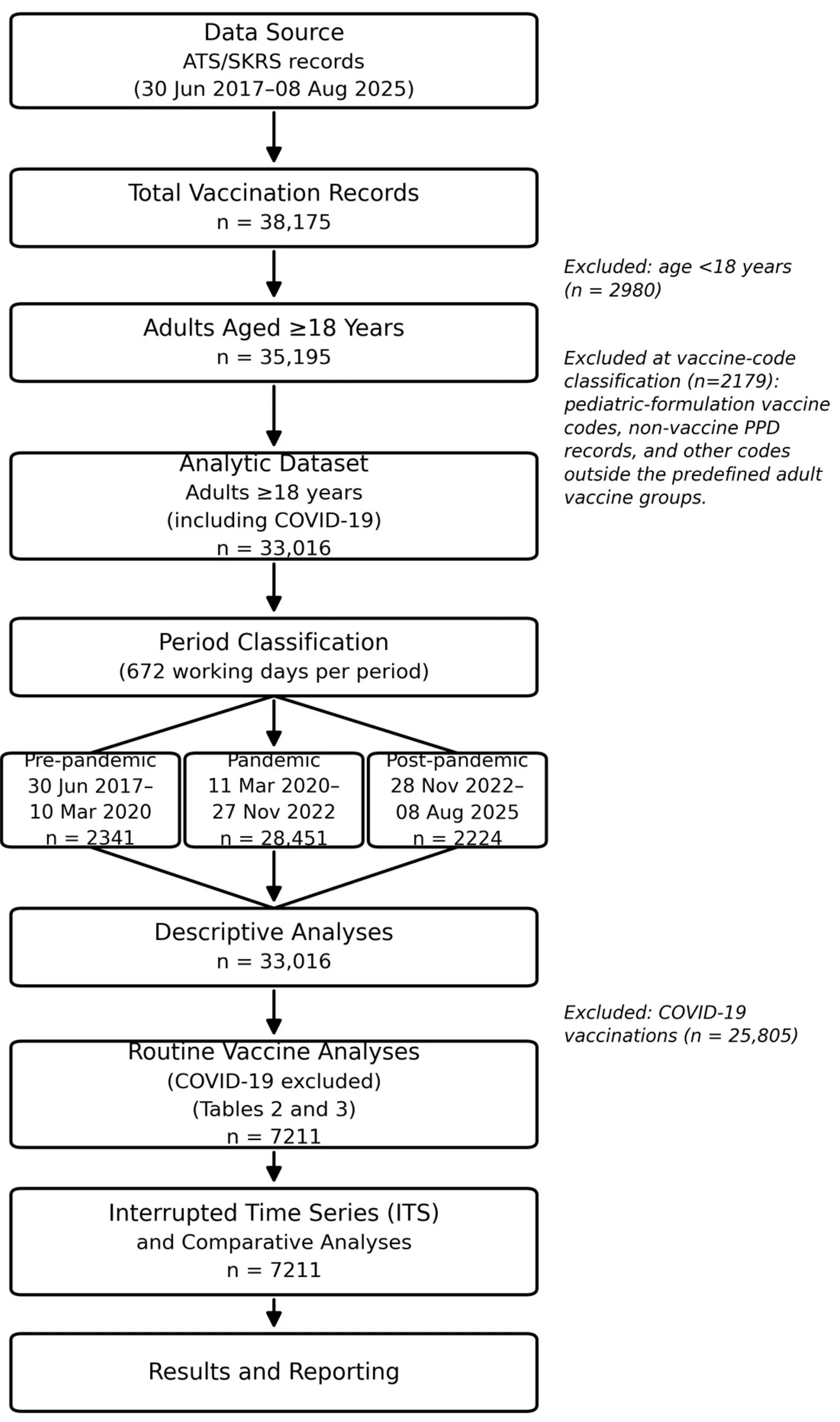
Study Flow Diagram and Analytical Framework. ATS: Vaccine Tracking System; SKRS: Health Coding Reference Server. Adults aged ≥18 years were included. Study periods were standardized to 672 working days each. COVID-19 vaccinations were excluded before routine vaccine analyses. After exclusion of records from individuals aged <18 years, an additional 2179 records were excluded at vaccine-code classification because they comprised pediatric-formulation vaccine codes recorded in adults, non-vaccine PPD records, or other codes outside the predefined adult vaccine groups. Records represent vaccination events rather than unique individuals; repeated valid vaccination events from the same individual were retained.

Data were obtained from the Health Coding Reference Server (SKRS), the national administrative health-data infrastructure of the Turkish Ministry of Health, integrated with the Vaccine Tracking System (ATS), the national electronic immunization registry [24,25]. All vaccination records generated during the study period for individuals aged ≥18 years at the time of vaccination and registered at one of the nine participating family medicine units were eligible for inclusion. Each record represented a vaccination event rather than a unique individual; therefore, the same individual could contribute multiple records if they received different vaccines or multiple doses during the study period. The study did not use a fixed cohort defined at a single baseline date; eligibility was determined at the time of each vaccination event. Accordingly, the analyses reflect recorded vaccination activity over time rather than population-based vaccination coverage. Of 38,175 total vaccination records, 35,195 belonged to adults ≥ 18 years. A further 2179 records were excluded at vaccine-code classification because they did not meet the prespecified adult vaccine-group definitions used in the analysis; these included pediatric-formulation vaccine codes recorded in adults, non-vaccine PPD records, and other codes not assignable to the predefined adult vaccine groups. The remaining 33,016 records constituted the analytic sample for descriptive analyses. Excluding a further 25,805 COVID-19 vaccination records yielded 7211 routine vaccination records used in the vaccine-group and ITS analyses, with the exclusion cascade summarized in Figure 1. For the purposes of this study, “routine non-COVID-19 vaccination” was used as an operational term for all non-COVID-19 vaccine administrations recorded in participating primary care units, including both scheduled preventive vaccines and indication-driven vaccinations such as rabies post-exposure prophylaxis. RSV and recombinant herpes zoster vaccines were not included in the longitudinal analyses because they became available in Türkiye only near the end of the observation period, precluding meaningful comparison across the pre-pandemic, pandemic, and post-pandemic periods.’

### 2.4. Statistical Analysis

Data were analyzed using IBM SPSS Statistics v25.0 and Python statsmodels (v0.14). The unit of observation was the monthly vaccination count across a 97-month series (pre-pandemic: 32; pandemic: 33; post-pandemic: 32 months); two partial boundary months (30 June 2017, 8 August 2025) were excluded to avoid biasing rate estimates, though record-level data span 99 calendar months. Because COVID-19 vaccines dominated the pandemic period, all primary analyses used the COVID-19-excluded dataset (*n* = 7211).

Primary analysis—ITS regression: ITS regression is a quasi-experimental method for evaluating intervention impact on time series data [26]. The immediate level (D1) and slope (Tpan) change at pandemic onset, and the corresponding level (D2) and slope (Tpost) change at pandemic end, were estimated using segmented negative binomial regression with log(monthly working days) as offset and a pre-pandemic trend parameter (t); counterfactual trends were estimated by setting pandemic indicators to zero. Counterfactual trajectories represent model-based extrapolations of the pre-pandemic trend and should therefore be interpreted as statistical reference trajectories rather than clinically attainable vaccination volumes, particularly when the pre-pandemic slope was steep. For Td, recovery was additionally quantified at the final modeled observation by comparing the fitted vaccination rate with the counterfactual rate expected under continuation of the pre-pandemic trajectory; the absolute and relative differences between these estimates were calculated. Poisson regression was initially considered for the count outcomes; however, substantial overdispersion was identified in the vaccine-group series. Accordingly, negative binomial regression was selected as the primary ITS model, with results reported as incidence rate ratios (IRRs) and 95% confidence intervals. The time variable was specified in months; for interpretability, slope coefficients were annualized and reported as per-year IRRs by exponentiating 12 times the corresponding monthly regression coefficient [IRR = exp(12β)]. Immediate level-change IRRs (D1 and D2) were not annualized. Negative binomial regression was retained as the primary model because overdispersion was a prominent feature of the count outcomes, whereas the HAC-robust Poisson model was used specifically to assess whether statistical inference was robust to residual serial dependence. Because substantial positive residual autocorrelation was identified (Durbin-Watson: 0.06–0.59), Newey-West HAC-robust Poisson regression (maxlags = 6) was applied as sensitivity analysis. All five primary negative binomial vaccine-group models, including influenza, are reported in full for transparency; however, influenza-specific inference from the primary model was interpreted cautiously because of pronounced seasonality and residual dependence. Center-level analyses were performed using 291 center-month observations (97 months × three centers), with Gaziosmanpaşa (Center C) as the reference center. The primary center-adjusted model included center as a main effect and therefore estimated overall differences in recorded vaccination activity between centers. In an additional exploratory analysis, center-by-interruption interaction terms were introduced for the pandemic-onset level change (D1), within-pandemic slope change (Tpan), pandemic-end level change (D2), and post-pandemic slope change (Tpost) to assess whether pandemic-associated temporal changes differed between centers. The same model specification was applied in both period-definition sensitivity analyses to assess whether the principal findings were dependent on equal-period standardization or the selected pandemic endpoint. Residual autocorrelation structure was further examined using autocorrelation (ACF) and partial autocorrelation (PACF) functions. These diagnostics indicated predominantly short-lag dependence, supporting the use of a six-lag Newey–West HAC specification. Calendar seasonality was additionally assessed using harmonic terms. For influenza, which showed pronounced seasonal variation, a seasonality-adjusted additive linear ITS model was fitted using annual and semiannual Fourier terms [sin(2πt/12), cos(2πt/12), sin(4πt/12), and cos(4πt/12)]. Because residual serial dependence persisted after seasonal adjustment, Newey–West HAC-robust standard errors (maxlags = 6) were used for inference in this model. To assess the sensitivity of the primary findings to the log-link functional form and the resulting exponential counterfactual extrapolation, additive linear segmented ITS models of vaccination rates per 1000 working days were also fitted using the same interruption terms (t, D1, Tpan, D2, and Tpost). Corresponding center-specific additive models were evaluated with Newey–West HAC-robust standard errors (maxlags = 6). These analyses were treated as functional-form sensitivity analyses and did not replace the primary negative binomial models.

The vaccine-specific ITS models were considered the primary analyses. Given the multiple level- and slope-change parameters estimated across vaccine groups, no formal multiplicity adjustment was applied to the ITS models; instead, borderline findings were interpreted cautiously in conjunction with effect estimates, confidence intervals, and consistency across sensitivity analyses.

Secondary analysis: Vaccine group distributions across periods were compared using Pearson chi-square (χ^2^) tests with Bonferroni-corrected post hoc comparisons (*p* < 0.017) and Cramér’s V effect sizes (<0.10 negligible; 0.10–0.29 small; ≥0.30 moderate); significance was set at *p* < 0.05. Age-group distributions across periods were additionally compared for pneumococcal and influenza vaccination events using Pearson χ^2^ tests with Cramér’s V; when the omnibus test was significant, Bonferroni-corrected pairwise comparisons were performed using *p* < 0.017.

### 2.5. Ethics

The study was approved by the Scientific Research Ethics Committee of Istanbul Prof. Dr. Cemil Taşcıoğlu City Hospital (Decision No. 196; 27 April 2026). The approved study protocol covered all three participating training family health centers and their nine family medicine units, each of which is affiliated with Istanbul Prof. Dr. Cemil Taşcıoğlu City Hospital; therefore, no subsequent expansion of the study scope or additional ethics amendment was required. Data extraction was performed only after ethics approval, and the study was conducted in accordance with the Declaration of Helsinki.

During the preparation of this manuscript, the authors used an AI-based writing assistant (DeepL) for language editing and formatting purposes only. The authors have reviewed and edited the output and take full responsibility for the content of this publication.

## 3. Results

### 3.1. General Characteristics

A total of 33,016 vaccination events recorded among adults aged ≥ 18 years across nine units were analyzed: 2341 (7.1%) pre-pandemic, 28,451 (86.2%) pandemic, and 2224 (6.7%) post-pandemic, with the pandemic-period share largely reflecting COVID-19 vaccination campaigns. Demographic characteristics of the routine non-COVID-19 vaccination events are presented in Table 2.

#### Unique Individuals and Repeated Vaccination Records

Because an individual could contribute more than one vaccination record during the study period, the counts reported above represent vaccination events rather than unique persons. Linking event-level records to national identity numbers (available for 32,063/33,016 events, 97.1%) identified 9607 unique individuals among the 33,016 vaccination events (mean 3.3 events/person): 929 individuals (9.7%) contributed exactly one event, 2721 (28.3%) contributed two events, and 5957 (62.0%) contributed three or more events. Among the 7211 routine (non-COVID-19) vaccination events (identity linkage available for 7175/7211 events, 99.5%), 3748 unique individuals were identified (mean 1.9 events/person): 2046 individuals (54.6%) contributed one event, 913 (24.4%) contributed two events, and 789 (21.1%) contributed three or more events. Repeated vaccination events were retained because individuals could legitimately contribute multiple records through multidose schedules, booster doses, repeat visits, or different vaccine types; no records were removed on the basis of repeated individual identifiers. Given this repeated-measures structure, chi-square comparisons of demographic distributions across periods (Table 2 and Table 3) describe the composition of vaccination events rather than assuming independence across individuals; this limitation is revisited in Section 4.

### 3.2. Routine Vaccine Distribution

Excluding COVID-19 vaccinations, 7211 routine vaccination events were analyzed (pre-pandemic: *n* = 2341; pandemic: *n* = 2659; post-pandemic: *n* = 2211). Vaccine group distribution differed significantly across periods with moderate effect size (χ^2^ = 888.6; df = 12; *p* < 0.001; Cramér’s V = 0.248), with significant Bonferroni-corrected pairwise differences for all three period pairs (Pre–Pandemic: χ^2^ = 539.5; V = 0.328; Pre–Post: χ^2^ = 258.5; V = 0.238; Pandemic–Post: χ^2^ = 397.4; V = 0.286; all *p* < 0.001) (Table 3).

### 3.3. Interrupted Time Series Analysis

**Figure 2:**
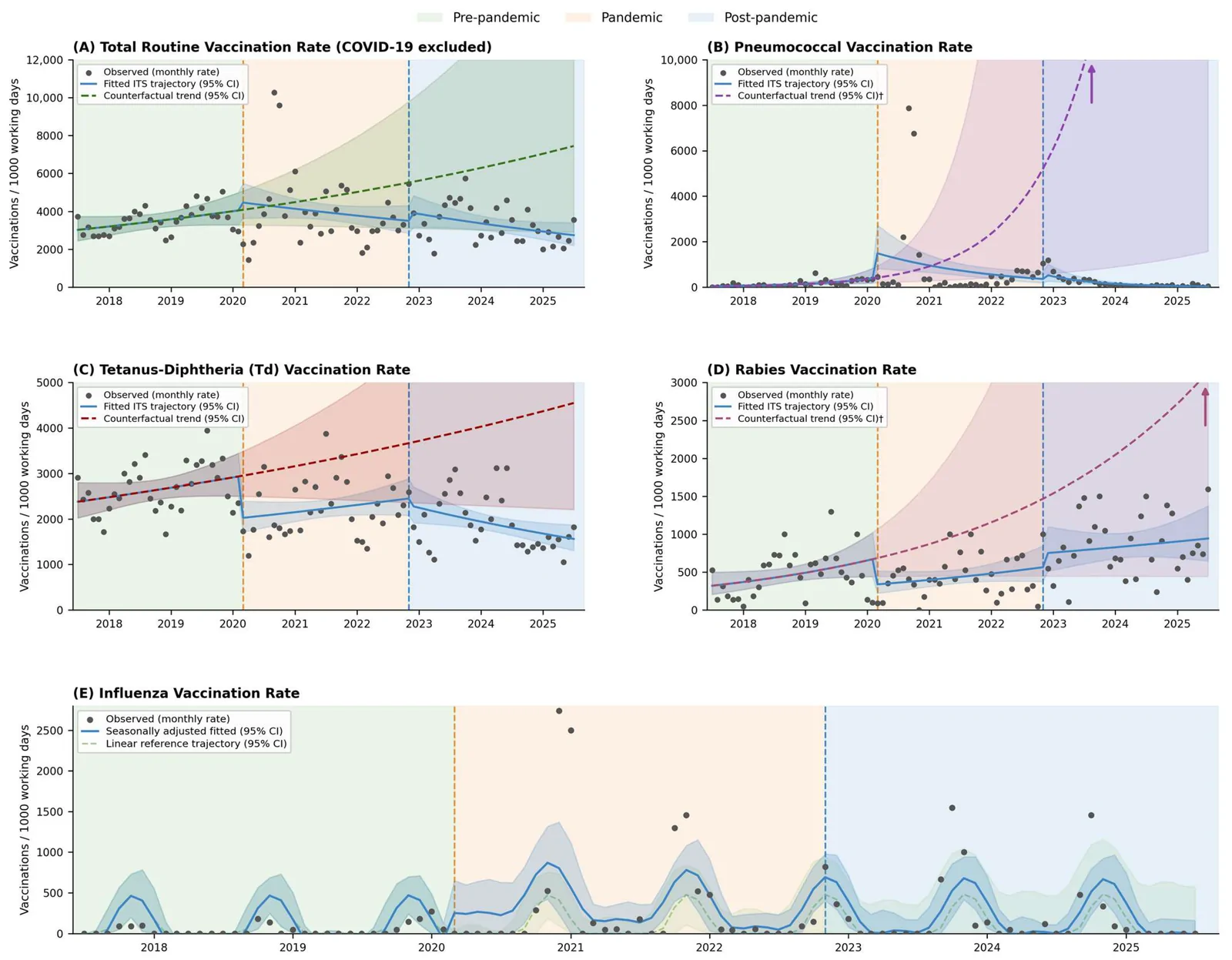
Interrupted time series (ITS) analysis of routine vaccination rates (June 2017–August 2025). Panels (**A**–**D**): primary negative-binomial ITS regression, replicated from the model underlying Table 4. Solid lines represent model-based fitted ITS trajectories, corresponding to monthly vaccination rates predicted by the respective models. Shaded areas represent 95% confidence intervals around the model-based fitted and counterfactual/reference trajectories. Observed monthly vaccination values are shown as individual points. For panels (**A**–**D**), dashed lines represent model-based counterfactual extrapolations of the pre-pandemic trend; in panel (**E**), the dashed line represents the linear reference trajectory. † In panels (**B**,**D**), the model-based counterfactual trajectory and/or its confidence interval extends beyond the displayed *y*-axis range; these counterfactual trajectories represent statistical reference extrapolations rather than clinically plausible future vaccination volumes. Panel (**E**): for influenza, the fitted and reference trajectories were derived from a seasonally adjusted additive linear ITS model including annual and semiannual Fourier terms; HAC-robust inference was used because residual serial correlation persisted. The pandemic-onset level change was not statistically significant after HAC correction (*p* = 0.086). Vertical dashed lines: pandemic onset (March 2020) and end (November 2022). Rate: vaccinations per 1000 working days. (**A**) Total routine vaccinations (COVID-19 excluded); (**B**) Pneumococcal; (**C**) Tetanus-Diphtheria (Td); (**D**) Rabies; (**E**) Influenza. *n* = 97 monthly observations.

The ITS analysis evaluated immediate level and slope changes at pandemic onset (March 2020) and pandemic end (November 2022) across 97 monthly observations. Results are presented in Table 4; temporal trends are shown in Figure 2.

Total routine vaccination: No significant immediate level change occurred at pandemic onset (IRR = 1.092; 95% CI: 0.809–1.475; *p* = 0.564). The negative binomial model suggested a within-pandemic slope decline (IRR = 0.816; 95% CI: 0.672–0.990; *p* = 0.039); however, this borderline finding was not confirmed by HAC sensitivity analysis (*p* = 0.218) and was therefore interpreted cautiously. No significant post-pandemic level (*p* = 0.433) or slope (*p* = 0.644) change occurred.

Tetanus-Diphtheria (Td): A significant immediate level decrease occurred at onset (IRR = 0.686; 95% CI: 0.541–0.869; *p* = 0.002). Within-pandemic slope did not change (*p* = 0.899); the post-pandemic slope continued to decline significantly (IRR = 0.805; 95% CI: 0.687–0.944; *p* = 0.008), indicating continued deterioration rather than evidence of post-pandemic recovery. At the final modeled observation (July 2025), the fitted Td vaccination rate was 1561.2 per 1000 working days (95% CI: 1305.4–1867.2), compared with a model-based counterfactual rate of 4549.6 per 1000 working days (95% CI: 2206.7–9380.2) under continuation of the pre-pandemic trajectory. The fitted rate was therefore 2988.4 per 1000 working days lower than the counterfactual estimate, corresponding to a 65.7% relative deficit.

Pneumococcal: A significant pre-existing upward trend was present (pretrend IRR = 2.583; 95% CI: 1.466–4.551; *p* = 0.001), with an additional level increase at onset (IRR = 3.589; 95% CI: 1.313–9.807; *p* = 0.013) and a sharp within-pandemic slope decline (IRR = 0.227; 95% CI: 0.114–0.449; *p* < 0.001). No significant post-pandemic level change was identified (*p* = 0.331).

Rabies: A significant level decrease occurred at onset (IRR = 0.495; 95% CI: 0.267–0.915; *p* = 0.025); post-pandemic level and slope changes were not significant (*p* = 0.344 and *p* = 0.589) (Figure 2D).

Influenza: Marked seasonal clustering produced substantial overdispersion in the primary negative binomial model (alpha = 89.78), resulting in very wide confidence intervals and limiting reliable inference from that specification. In the seasonally adjusted additive linear ITS model, annual and semiannual Fourier terms jointly indicated pronounced calendar seasonality. Model fit improved substantially after seasonal adjustment, and residual lag-12 autocorrelation decreased; however, residual serial dependence persisted. With HAC-robust inference, the estimated pandemic-onset level increase was not statistically significant (β = 459.52 vaccinations per 1000 working days; 95% CI: −64.54 to 983.57; *p* = 0.086), and no other pandemic-associated level or slope change was significant (Figure 2E).

The principal vaccine-specific findings remained consistent in both period-definition sensitivity analyses. When the full available pre-pandemic period was used without equalizing period lengths, the immediate and post-pandemic declines in Td, the pre-pandemic upward trend, pandemic-onset level increase, and within-pandemic decline in pneumococcal vaccination, and the pandemic-onset decline in rabies remained statistically significant. The same principal findings were retained when 5 May 2023 was used as the alternative pandemic endpoint. In both alternative specifications, the within-pandemic decline in total routine vaccination was significant in the negative binomial model but was not confirmed by the HAC sensitivity analysis. Thus, the principal vaccine-specific findings were not materially dependent on either equal-period standardization or the selected pandemic endpoint. Residual ACF/PACF diagnostics confirmed predominantly short-lag autocorrelation. In functional-form sensitivity analyses using additive linear segmented ITS models, the principal Td findings were retained, with a significant pandemic-onset level decrease and post-pandemic slope decline, and the pandemic-onset increase in pneumococcal vaccination also remained significant. Other pneumococcal temporal components and the pandemic-onset decline in rabies were more sensitive to model specification. In center-specific additive linear models with HAC-robust inference, none of the pandemic-associated level or slope changes (D1, Tpan, D2, or Tpost) was statistically significant. These findings support the robustness of the principal Td and pneumococcal pandemic-onset effects while indicating that some vaccine-specific and center-specific temporal estimates were dependent on model specification.

### 3.4. Pneumococcal and Influenza Vaccination Age Distribution

Because pneumococcal vaccination showed the most pronounced ITS signal, its age distribution was examined separately: no significant difference across periods was observed (χ^2^ = 2.4; df = 6; *p* = 0.880; V = 0.039), with vaccinations concentrated in the 45–64 year (~47%) and ≥65 year (36–41%) age groups throughout.

Influenza vaccination events also showed a significant shift in age distribution across the three periods (χ^2^ = 14.0, df = 6, *p* = 0.030; Cramér’s V = 0.130). The proportion of vaccination events recorded among adults aged ≥65 years increased from 13.8% before the pandemic to 33.1% in the post-pandemic period, whereas the proportion among adults aged 18–29 years declined from 31.0% to 10.3%. In pairwise comparisons, only the pre-pandemic versus post-pandemic comparison remained significant after Bonferroni correction (*p* = 0.013).

### 3.5. Center-Level Distribution

**Figure 3:**
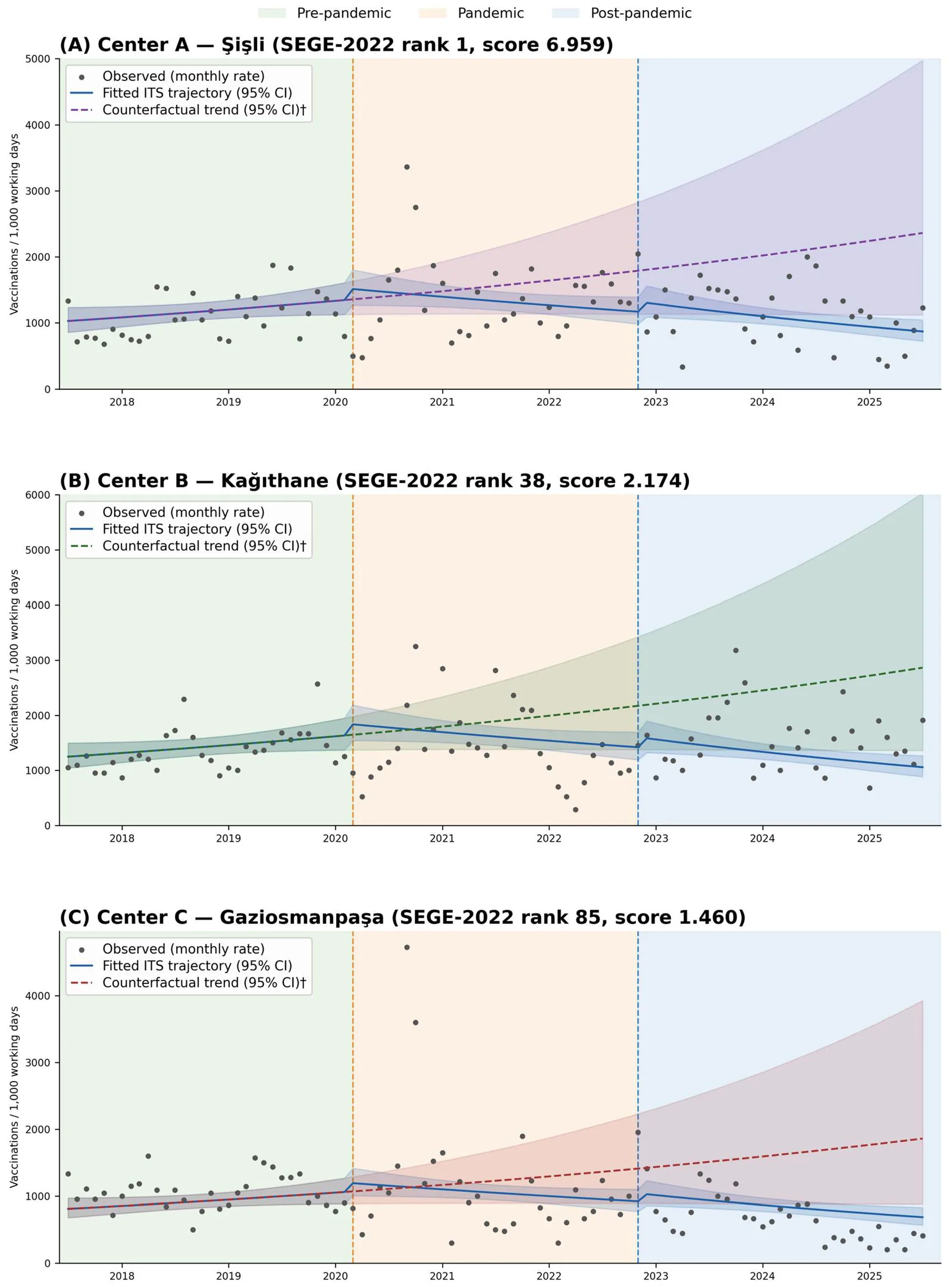
Center-level interrupted time series (ITS) analysis of routine vaccination rates. (**A**): Center A (Şişli, SEGE-2022 rank 1, score 6.959); (**B**): Center B (Kağıthane, SEGE-2022 rank 38, score 2.174); (**C**): Center C (Gaziosmanpaşa, SEGE-2022 rank 85, score 1.460). Solid line: model-based fitted ITS trajectory. Solid fitted trajectories represent monthly vaccination rates predicted by the corresponding ITS models. Shaded areas represent 95% confidence intervals around the model-based fitted and counterfactual trajectories. Observed monthly vaccination values are shown as individual points. † Dashed line: counterfactual trend (model-based extrapolation from the pre-pandemic slope). Vertical dashed lines: pandemic onset (March 2020) and end (November 2022). Rate: vaccinations per 1000 working days. COVID-19 excluded; 97 monthly observations per center (291 center-month observations in the center-adjusted model). The displayed center-specific trajectories are descriptive/model-based visualizations; formal between-center heterogeneity in pandemic-associated changes was assessed separately using center-by-interruption interaction terms.

The temporal distribution of routine vaccination records differed significantly by center (χ^2^ = 97.8; df = 4; *p* < 0.001; V = 0.082), broadly but not strictly aligned with SEGE-2022 ranks. With Center C (Gaziosmanpaşa) as reference, Center A (Şişli) showed a significantly higher vaccination rate (IRR = 1.269; 95% CI: 1.126–1.431; *p* < 0.001), and Center B (Kağıthane) showed the highest rate of all three (IRR = 1.539; 95% CI: 1.367–1.734; *p* < 0.001). Routine vaccination counts in Centers A, B, and C were 726, 902, and 713 (pre-pandemic), 926, 960, and 773 (pandemic), and 744, 1029, and 438 (post-pandemic), respectively (Figure 3).

In the exploratory center-by-interruption analysis, the omnibus test of all eight center-by-pandemic interaction terms indicated overall heterogeneity in temporal response across centers (likelihood-ratio χ^2^ = 44.47, df = 8, *p* < 0.001; Wald χ^2^ = 46.96, df = 8, *p* < 0.001). However, when the interruption components were evaluated separately, none showed statistically significant between-center heterogeneity in the corresponding two-degree-of-freedom joint tests (D1: *p* = 0.772; Tpan: *p* = 0.115; D2: *p* = 0.272; Tpost: *p* = 0.081). Thus, the exploratory model suggests overall center-level heterogeneity in pandemic-associated temporal patterns, but the available data do not clearly attribute this heterogeneity to a specific level or slope component.

## 4. Discussion

This multicenter ITS analysis of 97 monthly observations yields three principal findings: changes in routine adult vaccination across the pandemic period were vaccine-specific rather than uniform; pneumococcal vaccination increased, whereas Td and rabies declined. Influenza showed pronounced seasonal variation; after seasonal adjustment, the estimated pandemic-onset increase was not statistically significant using HAC-robust inference. Td alone showed continued post-pandemic deterioration rather than evidence of recovery during follow-up, and recorded routine vaccination activity per working day differed across centers without consistently aligning with district-level socioeconomic rank. These vaccine-specific patterns were robust to alternative temporal specifications, including use of the full available pre-pandemic period without equal-period restriction and use of the WHO PHEIC endpoint of 5 May 2023.

Total routine vaccination showed no abrupt level break at pandemic onset (D1 IRR = 1.092; *p* = 0.564), although a gradual within-pandemic decline was detected in the primary model (IRR = 0.816; *p* = 0.039) not confirmed by the HAC-robust sensitivity analysis (*p* = 0.218). This contrasts with WHO pulse-survey evidence of widespread disruption to essential health services in 2020 [8,9], a 170-country analysis documenting acute immunization service interruption [7], and reports that childhood DTP coverage had not returned to pre-pandemic levels globally as of 2023 [10,11]. It instead resembles US primary care findings, where non-COVID adult vaccination declines accumulated gradually over the first two pandemic years rather than as a single shock [12,13]. A plausible mechanism is that Turkish family health centers continued operating as first-contact facilities throughout the pandemic, preserving baseline capacity for routine immunization even as COVID-19 vaccination absorbed much clinical activity; this explanation is provisional given the slope finding was itself sensitivity-dependent.

Td vaccination followed a markedly different course: a significant immediate level decrease at onset (IRR = 0.686; 95% CI: 0.541–0.869) was followed by a continued post-pandemic slope decline (IRR = 0.805; 95% CI: 0.687–0.944), indicating persistent deterioration in recorded adult Td vaccination activity during follow-up. This interpretation was supported by the end-of-follow-up model predictions: the fitted Td vaccination rate in July 2025 was approximately 65.7% below the counterfactual rate projected from continuation of the pre-pandemic trajectory. However, the wide confidence interval around the counterfactual estimate reflects substantial uncertainty from long-term extrapolation and warrants cautious interpretation of the magnitude of this deficit. A plausible mechanism relates to how Td is used here: administered predominantly as post-injury prophylaxis rather than as a scheduled adult series, its decline may track reductions in trauma-related healthcare encounters reported elsewhere during the pandemic [27], though this link is indirect and should be read as a hypothesis. Because Td was the only vaccine showing a statistically significant continued post-pandemic decline, these findings support particular attention to Td vaccination activity in post-pandemic primary care.

Pneumococcal vaccination showed the most distinctive signal: an upward pre-pandemic trend (annual pretrend IRR = 2.583; 95% CI: 1.466–4.551) was followed by a sharp additional level increase at onset (IRR = 3.589; 95% CI: 1.313–9.807) and a rapid within-pandemic slope decline (annual slope-change IRR = 0.227; 95% CI: 0.114–0.449), with the age distribution of pneumococcal vaccination events remaining stable across periods (χ^2^ = 2.4; *p* = 0.880) and concentrated in the 45–64 and ≥65 year risk groups throughout. The magnitude of the immediate pneumococcal level increase should nevertheless be interpreted cautiously because of the wide confidence interval (IRR = 3.589; 95% CI: 1.313–9.807), indicating considerable uncertainty around the effect-size estimate. The strong pre-pandemic increase in pneumococcal vaccination should also be interpreted in the context of changes in adult pneumococcal vaccination practice in Türkiye. National risk-group vaccination guidance issued in 2016 strengthened pneumococcal vaccination recommendations for older adults and individuals with underlying risk conditions, with conjugate pneumococcal vaccination subsequently available through public health services [19]. Because our observation period began shortly thereafter, part of the steep pre-pandemic increase may reflect progressive implementation and clinical adoption of these recommendations rather than an indefinitely sustainable underlying trend. This may also partly explain the subsequent leveling of vaccination activity and reinforces the need for caution when extrapolating the pre-pandemic trajectory over the long term. This three-phase pattern is compatible with survey evidence that heightened concern about respiratory infection risk temporarily increased willingness to receive non-COVID vaccines before the effect receded [14,15]. Because vaccine indications were not recorded, this cannot be tested directly and is one plausible account among others, such as supply-side prioritization during the surge. The stable age distribution indicates that the age distribution of pneumococcal vaccination events remained broadly similar across periods; however, in the absence of clinical indications, comorbidity data, and vaccine eligibility information, this finding cannot establish whether vaccination was appropriately targeted to eligible risk groups. This transient surge suggests that acute public-health events may create opportunities to increase adult vaccination activity, although whether the observed vaccinations were appropriately targeted to eligible risk groups cannot be determined from the present data. The steep pre-pandemic pneumococcal trend also warrants caution, as long-term counterfactual extrapolation from the negative binomial model produced increasingly extreme projected rates.

Rabies vaccination showed an immediate decline at pandemic onset. In Türkiye, rabies vaccination in the general population is predominantly administered as post-exposure prophylaxis following potentially risky animal contact; therefore, vaccination activity is influenced by both the frequency of such exposures and subsequent healthcare utilization. The observed decline may thus reflect reduced opportunities for animal exposure during mobility restrictions, changes in healthcare-seeking behavior, or both. However, our dataset did not include animal-exposure events or clinically confirmed human rabies cases, precluding assessment of whether the decline in vaccination was accompanied by changes in exposure incidence or clinical rabies. Accordingly, the observed reduction should be interpreted as a change in recorded rabies vaccination activity rather than evidence of a change in rabies incidence. Influenza vaccination exhibited pronounced seasonality, and reliable inference was limited in the primary negative binomial ITS model. In the seasonally adjusted additive model, the estimated pandemic-onset increase did not reach statistical significance after HAC correction (*p* = 0.086), supporting a cautious interpretation of influenza-specific temporal changes.

For influenza vaccination, age distribution also differed across periods, with the proportion of vaccination events among adults aged ≥65 years increasing from 13.8% before the pandemic to 33.1% after the pandemic, while the proportion among those aged 18–29 years decreased from 31.0% to 10.3%. This shift suggests greater concentration of recorded influenza vaccination activity among older adults in the post-pandemic period; however, it should be interpreted cautiously given the small number of pre-pandemic influenza vaccination events and the pronounced seasonality of influenza vaccination.

Taken together, vaccines whose administration is opportunistic and linked to perceived infection risk or acute injury—pneumococcal, influenza, Td, and rabies—moved with the pandemic’s shifting infection concerns and care-seeking patterns, whereas changes in total routine vaccination, an aggregate across multiple indications, were less clearly defined and were sensitive to model specification. Pandemic-era monitoring of adult immunization should therefore track vaccine-specific indicators rather than an aggregate measure, which can mask offsetting increases and decreases.

Center-level analyses showed differences in recorded routine vaccination activity per working day across the three participating centers (χ^2^ = 97.8; *p* < 0.001), broadly but not strictly tracking each center’s SEGE-2022 rank. Center A (Şişli, rank 1 of 973 districts) showed a higher rate than the reference center (IRR = 1.269; *p* < 0.001). The more striking, and most novel, finding is that Center B (Kağıthane, rank 38) achieved the highest rate of the three (IRR = 1.539; *p* < 0.001), exceeding the center serving Türkiye’s highest-ranked district. These descriptive center-level differences should not be interpreted as evidence of an association between socioeconomic development and population vaccination uptake or coverage, because registered or vaccine-eligible population denominators were unavailable. Kağıthane represents a centrally located metropolitan district of Istanbul; however, according to the district-level SEGE classification used in this study, it occupied an intermediate socioeconomic position between Şişli and Gaziosmanpaşa. Therefore, its higher recorded vaccination activity should not be interpreted simply as an advantage attributable to higher socioeconomic development. Rather, the finding indicates a center-level difference in recorded vaccination activity whose underlying determinants cannot be identified from the present aggregate data. Thus, center-level vaccination patterns were not consistently aligned with district-level socioeconomic ranking. With only three centers, however, socioeconomic and center-specific organizational effects cannot be disentangled, and the observed differences should not be interpreted as evidence of an independent socioeconomic or organizational effect. Although the centers differed substantially in their absolute SEGE-2022 scores and rankings, all three districts ranked within approximately the top decile nationally (1st, 38th, and 85th of 973 districts). Therefore, these comparisons represent relative socioeconomic differences within a generally more-developed urban context rather than contrasts between highly developed and socioeconomically disadvantaged districts. Exploratory interaction analyses provided evidence of overall heterogeneity in pandemic-associated temporal patterns across centers, although no single interruption level or slope component showed clear between-center heterogeneity when tested jointly. Accordingly, the present data support differences in overall center-level vaccination activity and suggest heterogeneity in temporal response, but do not identify a specific phase of the pandemic as the source of these differences.

Several organizational factors could plausibly explain Kağıthane’s higher recorded vaccination activity per working day. Physician-level survey data from Istanbul indicate that reminder systems and physician awareness—rather than patient socioeconomic status per se—are the main drivers physicians identify for adult vaccination delivery [4], so differences in recall/reminder workflows could account for part of the gap. Patient panel size, workload, accessibility, age distribution, case mix, documentation practices, and center-specific vaccination workflows may differ across centers in ways not captured by district-level SEGE scores and could contribute to the observed variation. Single-center Turkish experience shows a dedicated, structured adult vaccination workflow can materially increase throughput independent of district socioeconomic development [28]. None of these hypotheses can be tested directly with the present ecological, center-level data; distinguishing between them would require individual-level data on physician practices and patient panels not available in this study.

Taken together, these findings argue for a shift in how adult immunization performance is monitored: tracking aggregate volume alone would have obscured both the Td shortfall and the Kağıthane–Şişli reversal documented here. Practical next steps include evaluation of strategies to strengthen Td vaccination activity and pilot assessment of potentially relevant organizational practices, rather than assuming socioeconomic investment alone would close between-center gaps.

### 4.1. Strengths

This study’s 97-month time series spanning 2017–2025 permits comprehensive evaluation of pre-pandemic, pandemic, and post-pandemic periods, and ITS analysis disentangles immediate from gradual pandemic effects. The study covers nine family medicine units across three training centers, enhancing transferability, and uses national SKRS/ATS data [24,25], providing routinely collected real-world vaccination records from participating primary care units. The primary analysis used equal working-day periods to facilitate temporal comparability; robustness was subsequently evaluated using the full available pre-pandemic period without equal-period restriction and the WHO PHEIC endpoint as an alternative pandemic end date. SEGE-2022 data enable ecological-level analysis of socioeconomic context.

### 4.2. Limitations

Center-level analyses used SEGE-2022 district-level data, which do not reflect individual socioeconomic characteristics; ecological fallacy cannot be excluded, and individual-level causal inference is not possible. Denominator data on registered population size were unavailable, so working days rather than per capita rates were used. Vaccinations administered outside the participating family health centers, including those delivered by other public or private healthcare providers, may not have been captured in the study dataset; therefore, the recorded vaccination activity should not be interpreted as complete ascertainment of all vaccinations received by the registered population. Changes in the size and demographic composition of the registered catchment populations over time could not be accounted for and may have influenced monthly vaccination counts. The analysis was event-based rather than an individual-level longitudinal analysis, and the same individual could contribute multiple vaccination events; therefore, event-level demographic comparisons should not be interpreted as independent person-level observations. Vaccine indications and individual risk factors were not recorded, precluding assessment of whether vaccine group changes reflected clinical need. As an aggregate ITS analysis, the study could not fully account for time-varying co-interventions or contextual changes, including changes in vaccine recommendations, eligibility criteria, reimbursement, vaccine supply, public campaigns, healthcare access, or center-level vaccination practices. Such factors may have contributed to the observed temporal changes and limit attribution of these changes specifically to the COVID-19 pandemic. The study was conducted in a single hospital-affiliated training FHC network in Istanbul; the three centers were selected for their variation in district-level socioeconomic development (SEGE-2022 ranks 1, 38, and 85 of 973 districts), central to the comparative aim, but this purposive selection limits generalizability to other cities, regions, or non-training settings. The study period extended through 8 August 2025 to maximize post-pandemic follow-up and preserve the equal-working-day design; this end date has no specific epidemiological significance. The choice of 27 November 2022 as the operational pandemic endpoint is inherently uncertain because the pandemic did not end at a single discrete time point; however, the principal findings were robust when the WHO PHEIC endpoint of 5 May 2023 was used in sensitivity analysis. Strong seasonal clustering in influenza (alpha = 89.78) limited reliable inference from the primary negative binomial ITS model. Although seasonally adjusted additive models incorporating annual and semiannual Fourier terms substantially reduced seasonal residual structure, residual serial dependence persisted; therefore, influenza-specific inference was based on HAC-robust estimates and interpreted cautiously. Substantial positive residual autocorrelation was detected (Durbin-Watson: 0.06–0.59), and HAC-robust sensitivity analyses indicated that some estimates were sensitive to residual dependence, including the within-pandemic slope for total routine vaccination. In addition, because the primary negative binomial models use a log link, extrapolation of a positive pre-pandemic slope can generate rapidly increasing counterfactual trajectories over long follow-up periods. These counterfactuals should therefore be regarded as statistical reference trajectories rather than forecasts of clinically attainable vaccination volumes, particularly for series with steep pre-pandemic trends. Additive linear functional-form sensitivity analyses produced less extreme counterfactual extrapolations and retained the principal Td changes and the pandemic-onset increase in pneumococcal vaccination, whereas other pneumococcal components and the pandemic-onset rabies decline were more sensitive to model specification. The registered FHC population may not fully represent the general population, and contributions from groups such as healthcare workers could not be separately evaluated. Because multiple vaccine-specific ITS parameters were evaluated, the possibility of type I error due to multiplicity cannot be excluded; accordingly, borderline findings should be interpreted cautiously and in conjunction with effect estimates, confidence intervals, and sensitivity analyses.

## 5. Conclusions

Changes in adult vaccination observed across the COVID-19 pandemic period in Istanbul primary care were vaccine-specific rather than uniformly negative: pneumococcal vaccination increased, whereas Td and rabies vaccination declined, and Td alone showed continued post-pandemic decline by the end of the study. Across the three centers, vaccination patterns were not consistently aligned with district-level socioeconomic ranking, indicating that the observed center differences should not be interpreted as a direct socioeconomic effect. These findings support surveillance frameworks that monitor vaccine- and unit-specific performance rather than aggregate vaccination volume alone.

## Figures and Tables

**Table 1:** SEGE-2022 socioeconomic development characteristics of study centers.

Center	District	SEGE-2022 Rank (of 973 Districts)	SEGE-2022 Score	Category
Center A	Şişli	1	6.959	Tier 1
Center B	Kağıthane	38	2.174	Tier 1
Center C	Gaziosmanpaşa	85	1.460	Tier 2

SEGE-2022: 2022 Socioeconomic Development Ranking of Districts. Turkish Ministry of Industry and Technology, Directorate General of Development Agencies; 2022. Centers are ordered from most (Center A) to least (Center C) developed.

**Table 2:** Demographic characteristics of routine (non-COVID-19) vaccination events by pandemic period (*n* = 7211).

Variable	Pre-Pandemic *n* = 2341	Pandemic *n* = 2659	Post-Pandemic *n* = 2211	Total *n* = 7211
Sex, *n* (%)				
Female	1558 (66.6)	1657 (62.3)	1497 (67.7)	4712 (65.3)
Male	783 (33.4)	1002 (37.7)	714 (32.3)	2499 (34.7)
Age group, *n* (%)				
18–29 years	1179 (50.4)	1066 (40.1)	1125 (50.9)	3370 (46.7)
30–44 years	857 (36.6)	783 (29.4)	684 (30.9)	2324 (32.2)
45–64 years	240 (10.3)	492 (18.5)	290 (13.1)	1022 (14.2)
≥65 years	65 (2.8)	318 (12.0)	112 (5.1)	495 (6.9)

Values are *n* (%), routine (non-COVID-19) vaccination events, COVID-19 excluded. Sex × Period: χ^2^ = 17.7; df = 2; *p* < 0.001; Cramér’s V = 0.050 (negligible effect); no significant difference between pre- and post-pandemic periods (*p* = 0.426). Age group × Period: χ^2^ = 291.6; df = 6; *p* < 0.001; V = 0.142 (small effect). Because individuals could contribute more than one vaccination event, these distributions describe vaccination-event characteristics rather than unique individual-level characteristics.

**Table 3:** Routine vaccine group distribution by pandemic period—COVID-19 excluded (*n* = 7211).

Vaccine Group	Pre-Pandemic *n* = 2341 (%)	Pandemic *n* = 2659 (%)	Post-Pandemic *n* = 2211 (%)	Total *n* = 7211 (%)
Tetanus-Diphtheria (Td)	1770 (75.6)	1489 (56.0)	1268 (57.3)	4527 (62.8)
Pneumococcal	98 (4.2)	556 (20.9)	124 (5.6)	778 (10.8)
Influenza	29 (1.2)	241 (9.1)	145 (6.6)	415 (5.8)
Rabies	314 (13.4)	296 (11.1)	575 (26.0)	1185 (16.4)
Hepatitis B (Adult)	47 (2.0)	56 (2.1)	33 (1.5)	136 (1.9)
MMR	40 (1.7)	19 (0.7)	54 (2.4)	113 (1.6)
Other *	43 (1.8)	2 (0.1)	12 (0.5)	57 (0.8)
Total	2341 (100)	2659 (100)	2211 (100)	7211 (100)

χ^2^ = 888.6; df = 12; *p* < 0.001; Cramér’s V = 0.248 (moderate effect). All period pairs significant at Bonferroni-corrected threshold (*p* < 0.017). * Other: rare groups with n < 50 (meningococcal, varicella).

**Table 4:** Interrupted time series analysis—negative binomial regression (*n* = 97 months, ref: pre-pandemic).

Parameter	Total Routine IRR (95% CI) *p*	Td IRR (95% CI) *p*	Pneumococcal IRR (95% CI) *p*	Rabies IRR (95% CI) *p*	Influenza ‡ IRR (95% CI) *p*
Pre-pandemic slope (pretrend)	1.119 (0.973–1.287) *p* = 0.114	1.084 (0.974–1.207) *p* = 0.139	2.583 (1.466–4.551) *p* = 0.001	1.333 (0.992–1.790) *p* = 0.056	1.732 (0.661–4.539) *p* = 0.264
Pandemic onset level change (D_1_)	1.092 (0.809–1.475) *p* = 0.564	0.686 (0.541–0.869) *p* = 0.002	3.589 (1.313–9.807) *p* = 0.013	0.495 (0.267–0.915) *p* = 0.025	5.341 (0.567–50.304) *p* = 0.143
Pandemic slope change (T_pan)	0.816 (0.672–0.990) *p* = 0.039	0.990 (0.851–1.152) *p* = 0.899	0.227 (0.114–0.449) *p* < 0.001	0.908 (0.608–1.356) *p* = 0.637	0.493 (0.111–2.185) *p* = 0.352
Pandemic end level change (D_2_)	1.129 (0.833–1.531) *p* = 0.433	0.940 (0.737–1.199) *p* = 0.619	1.602 (0.620–4.144) *p* = 0.331	1.319 (0.744–2.338) *p* = 0.344	1.183 (0.093–15.082) *p* = 0.897
Post-pandemic slope change (T_post)	0.955 (0.785–1.162) *p* = 0.644	0.805 (0.687–0.944) *p* = 0.008	0.524 (0.275–1.000) *p* = 0.050	0.902 (0.621–1.310) *p* = 0.589	0.817 (0.147–4.543) *p* = 0.817

IRR: incidence rate ratio; CI: confidence interval; slopes expressed as per-year IRR; all models include log(monthly working days) as offset. Negative binomial regression was the primary analysis; HAC-robust Poisson regression (maxlags = 6) served as a sensitivity analysis for residual serial dependence. The total routine Tpan estimate was not confirmed by HAC-robust inference (negative binomial *p* = 0.039 vs. HAC-Poisson *p* = 0.218). Additional additive linear functional-form sensitivity analyses are reported in Section 3. ‡ Influenza: Pronounced seasonality and overdispersion (alpha = 89.78) limited inference from the primary negative binomial model; therefore, influenza-specific inference was based on the seasonality-adjusted additive linear ITS model with annual and semiannual Fourier terms and HAC-robust standard errors, as reported in Section 3 and Figure 2E.

## Data Availability

The data presented in this study were obtained from the Turkish Ministry of Health SKRS/ATS national database. Access to these data is subject to institutional approval and may be requested from the corresponding author.

## Abbreviations

The following abbreviations are used in this manuscript:

ITS	Interrupted Time Series
SKRS	Health Coding Reference Server (Sağlık Kodlama Referans Sunucusu)
ATS	Vaccine Tracking System (Aşı Takip Sistemi)
SEGE-2022	2022 Socioeconomic Development Ranking of Districts
FHC	Family Health Center
Td	Tetanus-Diphtheria
MMR	Measles-Mumps-Rubella
IRR	Incidence Rate Ratio
CI	Confidence Interval
HAC	Heteroskedasticity and Autocorrelation Consistent
D1, D2	Level change at pandemic onset (D1) and end (D2)
Tpan, Tpost	Slope change during the pandemic (Tpan) and post-pandemic (Tpost) periods
WHO	World Health Organization
